# Mechanistic and Molecular Dynamics Studies Reveal that Increased Loop 3 Mobility Alters Substrate Capture in an NADH:Quinone Oxidoreductase

**DOI:** 10.1021/acs.biochem.5c00559

**Published:** 2025-12-13

**Authors:** Benjamin D. Dratch, Daniel Ouedraogo, Jacob Ball, Donald Hamelberg, Giovanni Gadda

**Affiliations:** α Department of Chemistry, 1373 Georgia State University, Atlanta, Georgia 30302-3965, United States; β Department of Biology, 1373 Georgia State University, Atlanta, Georgia 30302-3965, United States; γ The Center for Diagnostics and Therapeutics, 1373 Georgia State University, Atlanta, Georgia 30302-3965, United States

## Abstract

Dynamic protein loops can act as molecular gates that stabilize enzyme–substrate complexes, yet the underlying motions are poorly defined. Here, we dissect the role of loop 3 in an NADH:quinone oxidoreductase (NQO, UniProt Q9I4V0) from *Pseudomonas aeruginosa* PA01 in governing substrate binding and catalysis. Previous mechanistic and structural studies proposed that loop 3 fluctuations regulate substrate binding; however, an associated atomic-level understanding of the conformational changes is lacking. We probe the role of loop 3 dynamics in substrate capture and catalysis by mutating conserved P78 to glycine, which perturbs the gate rigidity. Steady-state kinetics with NQO-P78G and NQO-WT at varying concentrations of NADH and coenzyme Q_0_ established a 3.5-fold decrease in the *K*_CoQ0_ value, a 2.0-fold reduction in the *k*_cat_ value, and a 1.8-fold increase in the *k*_cat_/*K*_CoQ0_ value. The anaerobic reductive half-reaction of NQO-P78G with NADH yielded a ≤3.5-fold decrease in the *k*_red_ value and an estimated 80-fold increase in the *K*_d_ value compared to NQO-WT. Molecular dynamics simulations of ligand-free NQO-P78G and NQO-WT suggest that the P78G mutation disrupts interdomain interactions, allowing loop 3 to sample more open conformations. The combination of mechanistic and computational experiments suggests that more open gate conformations minimally promote access of the smaller coenzyme Q_0_ substrate to the active site. In contrast, the bulkier NADH substrate is less likely to associate, as the more open conformations prevent key interactions with NQO gate residues from forming. These results build on previous studies with NQO by demonstrating that altering loop 3 gate rigidity modulates substrate binding.

## Graphical abstract

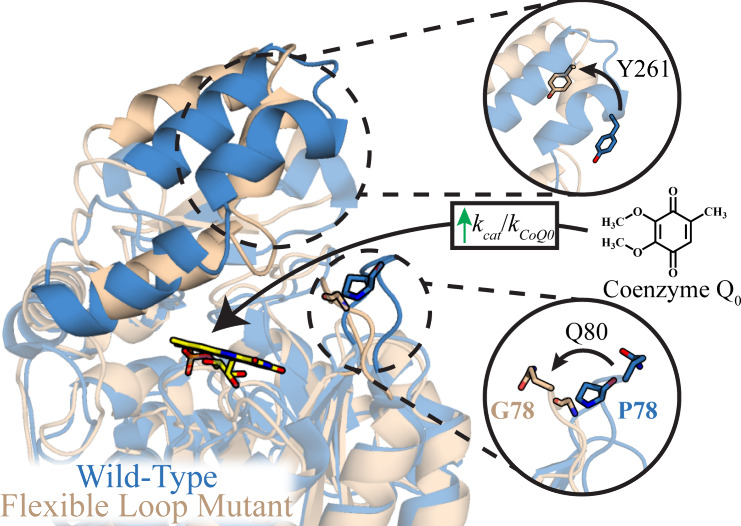

## Introduction

Enzymes are biological macromolecules that require a series of dynamic motions to maintain efficient catalytic rates and sustain life.^1–3^ Dynamics ranging from large domain motions to small side chain fluctuations, and even subatomic vibrations, modulate the degree of stiffness and flexibility within enzyme active sites.^4–9^ These regulated motions are required to achieve a preorganized active site environment, enabling them to facilitate the highly efficient and specific chemical reactions found in nature.^10^ Gates are a type of flexible protein region that adopts open and closed conformations to influence protein dynamics and modulate turnover rates by restricting the access of substrates, products, ions, or solvents to the interior of an enzyme.^1,10–13^ Furthermore, the chemical properties and steric hindrances of gate residues allow for a high level of control over the substrate specificity of an enzyme.^10,14^ As a result, when designing biocatalysts, modifications aimed at enhancing turnover rates or altering substrate specificity frequently target gating residues.^1,10,15^ For example, the haloalkane dehalogenase DhaA from *Rhodococcus rhodochrous* utilizes active site tunnels to detoxify the industrial compound 1,2,3-trichloropropane.^16^ By generating a variant with the gate mutations I135F, C176Y, V245F, L246I, and Y273F, DhaA achieved a 32-fold increase in the *k*_cat_ and a 26-fold increase in the *k*_cat_/*K*_m_ values by preventing water molecules from entering the active site and subsequently switching the rate-limiting step from a chemical step to product release.^16^

Enzymology studies often identify the role of side chains and cofactors during turnover by evaluating the rates of enzyme complex formation and chemical processes to elucidate enzyme mechanisms and engineer more efficient variants.^17^ These experiments provide crucial information about an enzyme’s catalytic abilities; however, they cannot describe the dynamic motions that play an important role in enzyme turnover. In recent years, an influx of protein investigations has demonstrated a clear link between conformational dynamics and protein function, leading to an increased interest in the functional implications of protein dynamics.^10^ Specifically, the fluctuations of protein loops, or lack thereof, facilitate vital conformational changes necessary for efficient enzyme catalysis and substrate binding. For example, in the protein tyrosine phosphatase (PTP) family, YopH and PTP1B share a similar overall structure and active site configuration. Yet, YopH has a 32-fold higher *k*_cat_ value because the exchange rate between the WDP-loop open and closed conformations is 47-fold higher in YopH than in PTP1B.^18,19^ In the cytochrome P450 TxtE, replacing the F/G loop residue H176 with a tyrosine or tryptophan shifts gate dynamics to sample mostly closed-lid conformations and alters the regioselectivity of TxtE to produce 5-nitro-tryptophan instead of the naturally occurring product, 4-nitro-tryptophan.^20^ For the indole-3-glycerol phosphate synthase TrpC, catalysis is rate-limited at the ring-closure step; however, the rate-limiting step can be switched to a dehydration step by slowing loop 1 dynamics from a picosecond-to-millisecond timescale through alanine mutations.^21–23^ Recently, a kinetic investigation with NADH:quinone oxidoreductase (NQO, UniProt Q9I4V0) from *Pseudomonas aeruginosa* (*P. aeruginosa*) PAO1 demonstrated that mutating the gating residue Q80 to a glycine, leucine, or glutamate decreased the binding affinity of NADH by ≥25-fold without altering the rate-limiting step or the rate of product release.^24^ The study proposed that the Q80 mutations affected the binding affinity for NADH by destabilizing the closed gate conformation. However, this hypothesis has yet to be validated, thus demonstrating the importance of analyzing protein dynamics in concert with mechanistic studies to reveal the full story of enzyme turnover.

NQO is an FMN-dependent enzyme that utilizes NADH as a reducing substrate to detoxify a variety of quinones through a ping-pong bi-bi steady-state kinetic mechanism (Scheme 1).^24,25^ The physiological role of NQO is unknown; however, previous bioinformatic investigations suggest that the enzyme maintains a ratio of [NAD^+^]/[NADH] that is favorable for the β-oxidation pathway in *P. aeruginosa*.^25–28^ Structurally, NQO consists of a TIM-barrel domain (M^1^-P^211^ and E^299^-V^328^) and an extended domain (I^212^-D^298^), where a hinge region connects the two domains to form the active site pocket.^29^ Crystal structures with and without NAD^+^ bound in the active site of NQO reveal that loop 3 of the TIM-barrel domain (residues 75–86) acts as a gate by moving 5.5 Å toward the active site upon ligand binding (Figure 1).^29^ Previous bioinformatic studies revealed that residues T75 and P78 of loop 3 are highly conserved within the second structural motif of a novel family of NQOs (^66^TXXPFGVNXThhP^78^, where h is any hydrophobic residue and X is any residue).^25,30^ Loop 3 contains three proline residues thought to facilitate the gate’s transition between catalytically active and inactive conformational states by modulating structural rigidity, yet P78 is the only one conserved in the second structural motif. Herein, we hypothesize that the structural rigidity provided by P78 plays an essential role in modulating gate conformations and, therefore, the catalytic cycle of NQO.

**Scheme 1:**
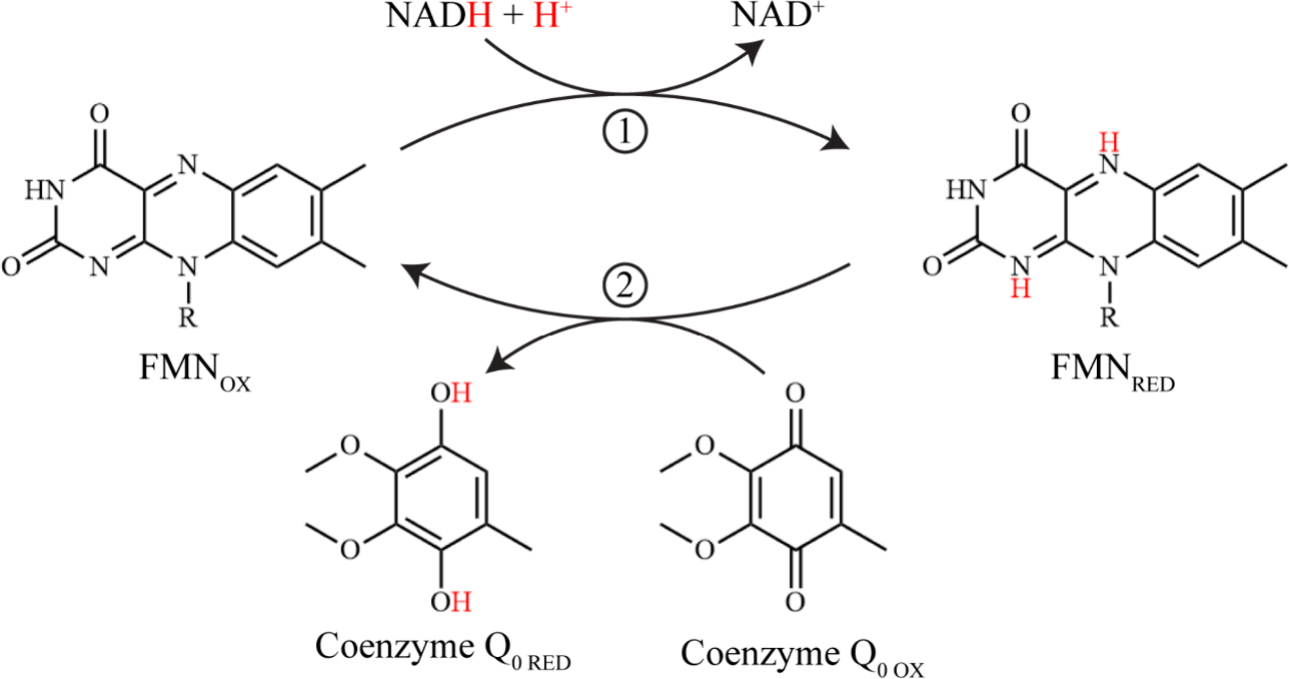
Reaction Mechanism of NQO with Coenzyme Q_0_ as an Oxidizing Substrate

**Figure 1:**
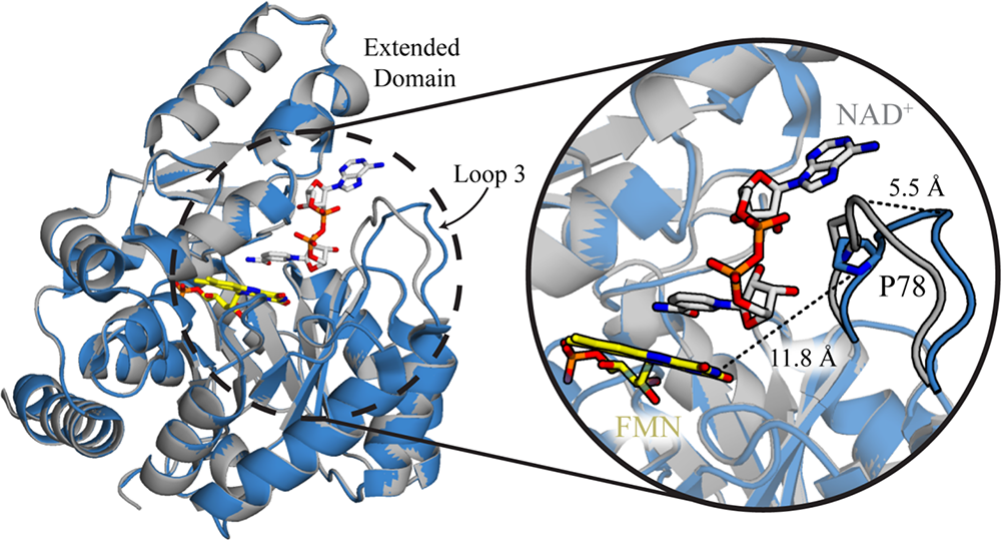
The overall structure of NQO is shown in the ligand-free form (blue, PDB: 2GJL) and the NAD^+^-bound form (gray, PDB: 6E2A). The gate region is magnified to better depict the conformational change at loop 3 following substrate binding. Dashed lines show the distance spanned by the α-carbon of Q80 following gate closure or the distance between the α-carbon of P78 and N3 of FMN. Yellow and silver sticks depict the FMN and NAD^+^ carbons, respectively.

In this study, the role of loop 3 dynamics in facilitating substrate binding in NQO was investigated by mutating the conserved P78 to a glycine (NQO-P78G) to reduce the backbone rigidity of loop 3. The α-carbon of P78 is 11.8 Å from N3 of the FMN cofactor in NQO (Figure 1). Thus, we hypothesized that mutating the conserved P78 would have a minimal effect on the active site microenvironment of NQO. In contrast, the P78G mutant is expected to destabilize the dynamics of loop 3 and alter the rate of enzyme–substrate association. Herein, a combination of UV–visible absorption spectroscopy, molecular dynamics (MD) simulations, and steady-state and rapid reaction kinetics was utilized to highlight the relationship between enzyme gate dynamics and catalysis in NQO.

## Experimental Procedures

### Materials

*Pfu* DNA polymerase was purchased from Stratagene (La Jolla, CA). A QIAprep Spin Miniprep Kit and a QIAquick PCR Purification Kit were from Qiagen (Valencia, CA). *Dpn*I, CutSmart Buffer, and a deoxynucleotide solution mix were obtained from New England Biolabs (Ipswich, MA). Oligonucleotides containing the P78G point mutation were purchased from Sigma Genosys (The Woodlands, TX). Isopropyl-1-thiol-β-d-galactopyranoside (IPTG) was ordered from Promega (Madison, WI). *Escherichia coli* (*E. coli*) strains DH5α and Rosetta­(DE3)­pLysS were purchased from Invitrogen Life Technologies (Grand Island, NY) and Novagen (Madison, WI), respectively. A HiTrap Chelating HP 5 mL affinity column and prepacked PD-10 desalting columns were obtained from GE Healthcare (Piscataway, NJ). All other reagents used were of the highest purity commercially available.

### Site-Directed Mutagenesis and Purification of NQO-P78G

NQO-P78G was prepared using PCR with the pET20b­(+)/NQO plasmid as a template.^25^ The PCR product was purified using the QIAquick PCR Purification Kit, digested with *Dpn*I, and used to transform chemically competent DH5α cells via the heat shock method.^31^ The pET20b­(+)/NQO-P78G plasmids were subsequently extracted from DH5α cells, purified using a QIAquick Spin Miniprep Kit, and sequenced by Macrogen (Rockville, MD). NQO-P78G was expressed in *E. coli* strain Rosetta­(DE3)­pLysS and purified using methods previously described for NQO-WT.^25^ The purified NQO-P78G was stored at −20 °C in 20 mM sodium phosphate, pH 8.0, 100 mM sodium chloride, and 10% glycerol.

### UV–Visible Absorption Spectroscopy

UV–visible absorption spectra were recorded by using an Agilent Technologies (Santa Clara, CA) model HP 8453 diode-array spectrophotometer. The flavin to protein stoichiometry in NQO-P78G was determined by incubating the enzyme at 100 °C for 20 or 30 min in 20 mM potassium phosphate, pH 7.0, and 200 mM sodium chloride, followed by removal of the precipitated protein by centrifugation at 23,000*g*.^32^ The concentration of free flavin released from the denatured NQO-P78G was determined spectroscopically by using ε_450_ = 12,200 M^–1^ cm^–1^. The flavin content per NQO-P78G monomer was calculated by taking the ratio of free flavin over the total protein concentration, resulting in an experimentally determined extinction coefficient of ε_461_ = 11,900 M^–1^ cm^–1^ for FMN-bound NQO-P78G.^33^ The total protein concentration was determined using the Bradford method with bovine serum albumin as a standard.^34^

The UV–visible absorption spectra for NQO-P78G and NQO-WT were measured as a function of pH in 10 mM sodium phosphate, 10 mM sodium pyrophosphate, pH 8.0, and 100 mM sodium chloride at 15 °C. The pH was adjusted to a final value of 11.5 by adding 1 M sodium hydroxide in 10 μL increments while stirring. Following each addition of sodium hydroxide, the enzyme solution was allowed to equilibrate until no changes in the pH value and absorption spectra were observed, typically requiring a waiting period of 2–3 min. Resulting spectra were corrected for protein concentration below 325 nm using ε_461_ = 11,900 M^–1^ cm^–1^ for NQO-P78G and ε_461_ = 12,400 M^–1^ cm^–1^ for NQO-WT.^25^

### Molecular Dynamics Simulations

Multiple μs-long molecular dynamics (MD) simulations were performed on four systems, each containing an FMN cofactor: (i) NQO-P78G, (ii) NQO-WT, (iii) NQO-P78G with bound NAD^+^, and (iv) NQO-WT with bound NAD^+^. Initial coordinates for MD simulations were taken from the crystal structures of NQO-WT in the ligand-free form (PDB: 2GJL) or with NAD^+^ bound (PDB: 6E2A). MD simulations were performed using AMBER 16 with the AMBER ff14SB force field.^35,36^ Force field parameters for the FMN cofactor were taken from Sühnel and Schneider.^37^ NQO-P78G was prepared *in silico* by altering the amino acid sequence in the NQO-WT PDB files. The AmberTools xleap program was used to construct the appropriate system required for each MD simulation by building any missing side chain atoms.

Each system was solvated in a TIP3P octahedron box with each edge of the system at least 10 Å from each hexagonal face, along with chloride ions used to neutralize the system.^38,39^ Energy minimization involved 2000 steps of steepest descent followed by 3000 steps of conjugate gradient, where harmonic restraints held the position of the protein. Five rounds of energy minimization were performed, where the force constant of the positional restraint was gradually reduced from 500 to 0 kcal mol^–1^ Å^–2^. Each system was then heated from 100 to 300 K within 500 ps under NVT periodic conditions with a 1 fs time step. Five rounds of heating were then performed, where the force constant of the restraint was set to 500, 300, 100, 50, and 5 kcal mol^–1^ Å^–2^. A 1 ns equilibration step was performed with a 2 fs time step where the whole system was allowed to move freely. All simulations were then run under NPT (300 K, 1 bar) periodic conditions, where the temperature was regulated using the Langevin thermostat using a 1 ps^–1^ collision frequency. Simultaneously, the pressure was controlled using a Monte Carlo barostat with a coupling constraint of 1 ps. Long-range nonbonded electrostatic interactions were evaluated using the particle mesh Ewald (PME) method with a cutoff of 9 Å.^40^ All bonds involving hydrogen atoms were constrained using the SHAKE algorithm.^41^ The MD simulations for (ii) NQO-WT and (iv) NQO-WT with NAD^+^ were carried out for 1 μs. In contrast, (i) NQO-P78G and (iii) NQO-P78G with NAD^+^ were simulated for 3.5 and 1.5 μs, respectively. Subsequent analyses were conducted using the last 0.8 μs of the trajectories for (ii) NQO-WT, (iii) NQO-P78G with NAD^+^, and (iv) NQO-WT with NAD^+^, as well as the last 1.6 μs of the (i) NQO-P78G trajectory.

### Principal Component Analysis and Distance Calculations

Principal component analysis (PCA) was applied to all four systems using the CPPTRAJ module of AMBER 16.^42^ Structures were superimposed at the backbone atoms (N, Cα, C, and O) from the first frame of the ligand-free NQO-WT simulation. The variance-covariance matrix characterizing correlated internal backbone motions was calculated and diagonalized to obtain eigenvectors and eigenvalues. Eigenvectors were projected back onto all four systems’ trajectories to obtain the principal components (PCs), which describe the structural variance between the simulations captured at each PC. The top two PCs (PC1 and PC2) captured the most significant structural variance between the simulations and were projected onto a 2D PC plot using ggplot2 to analyze the interconformer relationship between trajectories.^43^ The motions captured by PC1 and PC2 were visualized using VMD.^44^ To measure the flexibility of the backbone atoms in NQO, the root-mean-square deviation (RMSD) was calculated and analyzed using CPPTRAJ, Bio3D, and R packages.^45,46^ The fluctuations of the gate in NQO were monitored by first superimposing structures onto the backbone atoms and then calculating the separation between the gate residues.

### Difference Contact Network Analysis

Residue–residue contacts were analyzed for NQO-P78G and NQO-WT following methods previously described.^47,48^ A contact was defined to have formed between residues if any heavy atoms of two residues, separated by three amino acids in the protein sequence (*i* to *i* + *n*, *n* ≥ 3), were within 4.5 Å from one another.^48,49^ The contact probability difference from NQO-WT to NQO-P78G was generated to assess how the contacts were affected by the P78G mutation. The changes in contact probability were further elucidated using the difference contact network analysis (dCNA) method.^50,51^ dCNA identifies and visualizes changes in residue–residue contacts between protein regions to better investigate the conformational variation between any two systems. The analysis and subsequent figures were prepared using Bio3D,^45,46^ VMD,^44,52^ and in-house R scripts.

### Steady-State Kinetics

Steady-state kinetic parameters were measured with an Agilent Technologies model HP 8453 diode-array spectrophotometer equipped with a thermostated water bath. The initial rates of the reaction for NQO-WT and NQO-P78G were determined with a final enzyme concentration of 100 nM and varying concentrations of NADH and coenzyme Q_0_ (CoQ_0_) for each enzyme in 20 mM potassium phosphate, pH 6.0, and 100 mM sodium chloride at 25 °C.^53^ Turnover of NQO-WT was analyzed by varying NADH over 30–100 μM and CoQ_0_ over 30–150 μM, where NQO-P78G varied NADH from 10 to 100 μM and CoQ_0_ from 2 to 10 μM. Solutions of CoQ_0_ were prepared in 100% ethanol and then added to the reaction mixture so that the final concentration of ethanol was 1%, thus preventing any possible deleterious effects of ethanol on enzyme activity. Reaction rates were measured by following NADH consumption at 340 nm using ε_340_ = 6220 M^–1^ cm^–1^.^54,55^ Enzymatic reactions were initiated by the addition of NQO-WT or NQO-P78G to the reaction mixture.

### Reductive Half-Reaction

The reductive half-reaction of NQO was investigated anaerobically and under pseudo-first-order conditions in a stopped-flow spectrophotometer, SF-61DX2 Hi-Tech KinetAsyst (Bradford on Avon, U.K.) thermostated at 25 °C. The reduction of enzyme-bound FMN via NADH was monitored by following absorbance changes at 461 nm in 20 mM potassium phosphate, pH 6.0, and 200 mM sodium chloride. Previously described protocols were utilized to ensure that the buffers, instrument, substrates, and enzymes employed in this study were fully anaerobic.^25^ NADH concentrations ranging from 90 to 500 μM and enzyme concentrations of 24 μM for NQO-WT and 30 μM for NQO-P78G were utilized to ensure that pseudo-first-order conditions were maintained by fully saturating the enzymes with NADH.

### Data Analysis

The data from the kinetic studies were fit with KaleidaGraph software (Synergy Software, Reading, PA), and the global analysis was carried out using the Kinetic Studio Software Suite Enzfitter (Hi-Tech Scientific, Bradford on Avon, U.K.). Steady-state kinetic parameters at varying concentrations of NADH (*A*) and CoQ_0_ (*B*) were determined by fitting the Michaelis–Menten equations to the initial rates of the reactions. Equation 1 describes a standard ping-pong bi-bi steady-state mechanism, while eq 2 accounts for substrate inhibition by NADH. Initial rates were calculated using the experimentally determined ratio of a reaction’s initial velocity (*v*_0_) and enzyme concentration (*e*). *K*_a_ represents the Michaelis–Menten constant for NADH, *K*_b_ represents the Michaelis–Menten constant for CoQ_0_, *k*_cat_ is the enzyme turnover number at saturated concentrations for both substrates, and *K*_is_ is the inhibition constant that describes the binding of NADH to the reduced enzyme, yielding a dead-end complex.

$$\frac{v_{o}}{e}=\frac{ABk_{\mathrm{cat}}}{AK_{b}+BK_{a}+AB}$$ 1

$$\frac{v_{o}}{e}=\frac{ABk_{\mathrm{cat}}}{K_{a}B+K_{b}A(1+\frac{A}{K_{\mathrm{is}}})+AB}$$ 2

Stopped-flow traces were fit to eq 3, which defines a double-exponential process. *A* represents the absorbance at 461 nm at any given time *t*, *B*_1_ and *B*_2_ are the amplitudes of the decrease in absorbance, *k*_obs1_ and *k*_obs2_ represent the observed first-order rate constants for the reduction of the enzyme-bound flavin at any given concentration of the substrate associated with the absorption changes at 461 nm, and *C* is an offset value accounting for the nonzero absorbance of the enzyme-bound reduced flavin at infinite time. The kinetic parameters of the reductive half-reaction were determined using eq 4, which defines a hyperbolic saturation of the enzyme with NADH with a *y*-intercept value of zero. Here, *k*_obs_ represents the observed first-order rate constant for the reduction of the enzyme-bound flavin at any given substrate concentration (*S*), *k*_red_ is the rate constant for flavin reduction at saturated concentrations of the substrate, and *K*_d_ represents the equilibrium constant for the dissociation of the enzyme–substrate complex into a free substrate and enzyme.

$$A=B_{1}\exp\left(-k_{\mathrm{obs}1}t\right)+B_{2}\exp\left(-k_{\mathrm{obs}2}t\right)+C$$ 3

$$k_{\mathrm{obs}}=\frac{k_{\mathrm{red}}S}{K_{d}+S}$$ 4

## Results

### UV–Visible Absorption Spectroscopy with NQO-P78G

To establish if the replacement of P78 with glycine altered the active site microenvironment in NQO, the UV–visible absorption spectrum of NQO-P78G was determined as a function of pH and compared to that of NQO-WT. The absorption spectrum for NQO-P78G at pH 8.0 revealed two excitation peaks at 370 and 461 nm, which are characteristic of oxidized flavoproteins (Figure 2A). As pH values increased from 8.0 to 11.5, the absorption spectrum of NQO-P78G was minimally affected, where a ≤2% change in absorption intensities and a ≤5 nm difference in wavelengths were observed for both peaks. The absorption spectra for NQO-WT were determined and compared to those of NQO-P78G, which revealed differences of ≤15% in the intensity of the 370 nm peak, ≤4% in the intensity of the 461 nm peak, and ≤2 nm in the wavelengths of both oxidized peaks from pH 8.0 to 11.5 (Figure 2B). The most considerable spectral changes resulting from the increase in pH were seen at 297 nm for both enzymes (Figure 2).^56^ However, a previous study with NQO demonstrates that the increase at 297 nm corresponds to the deprotonation of Y277, which does not perturb the flavin microenvironment in the presence of salt ions.^56^

**Figure 2:**
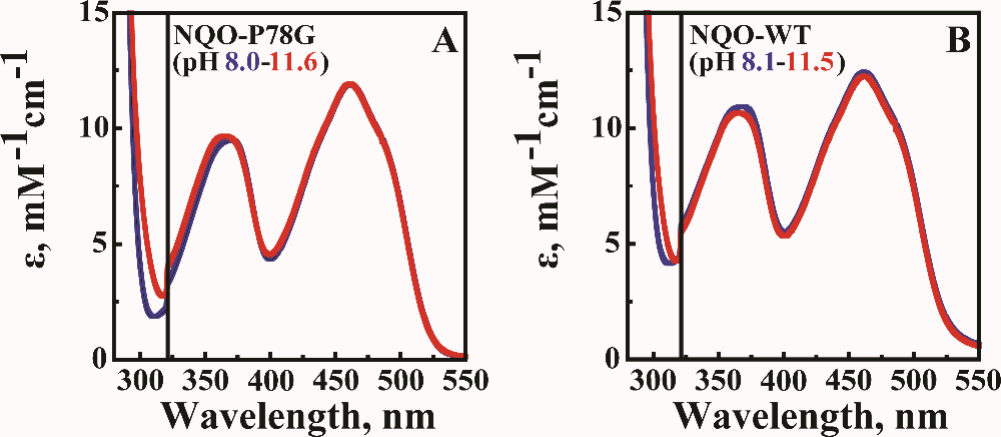
Absorption spectra for (A) NQO-P78G and (B) NQO-WT are shown as the pH increases from 8.0 (blue) to 11.5 (red). Extinction coefficient values were corrected for the protein absorption (≥320 nm) by adjusting for flavin binding. The FMN/enzyme stoichiometry is 0.7 for NQO-P78G and 0.8 for NQO-WT. To highlight the absorption from FMN, the largest peak at 297 nm is not fully shown. Spectra were recorded in 10 mM sodium phosphate, 10 mM sodium pyrophosphate, pH 8.0, 10 mM sodium chloride, and 20% glycerol at 15 °C.

### Backbone Dynamics of NQO-P78G

MD simulations were performed on NQO-P78G and NQO-WT, both with and without bound NAD^+^. The starting models for NAD^+^-free and NAD^+^-bound NQO simulations were taken from PDB structures 2GJL and 6E2A, respectively. Resulting trajectories were compared using PCA to investigate how the P78G mutation alters the fluctuations of NQO gate conformations (see Experimental Procedures for details). A PCA plot depicting all four NQO simulation trajectories at the top two PCs (PC1 and PC2) demonstrates that the trajectories sample well-defined conformational spaces, thus indicating that the analyzed backbone dynamics represent localized conformations (Figure 3A). Additionally, a scree plot was generated to determine the percentage of dynamics motions from the NQO simulations that were captured by each PC. In general, a scree plot is used to identify the most significant PCs by plotting the total variability of the system captured by each PC. This analysis established that PC1 and PC2 represent the dominant collective motions as they capture 67% of the total structural variance between all four systems, whereas PC3 onward each captures minimal variations (Figure 3B). The conformational states of ligand-free NQO-P78G and NQO-WT contain no similarities at PC1 and only a minor overlap at PC2 (Figure 3A). In contrast, significant overlap between the conformational states of NAD^+^-bound NQO-P78G and NQO-WT at both principal components is observed.

**Figure 3:**
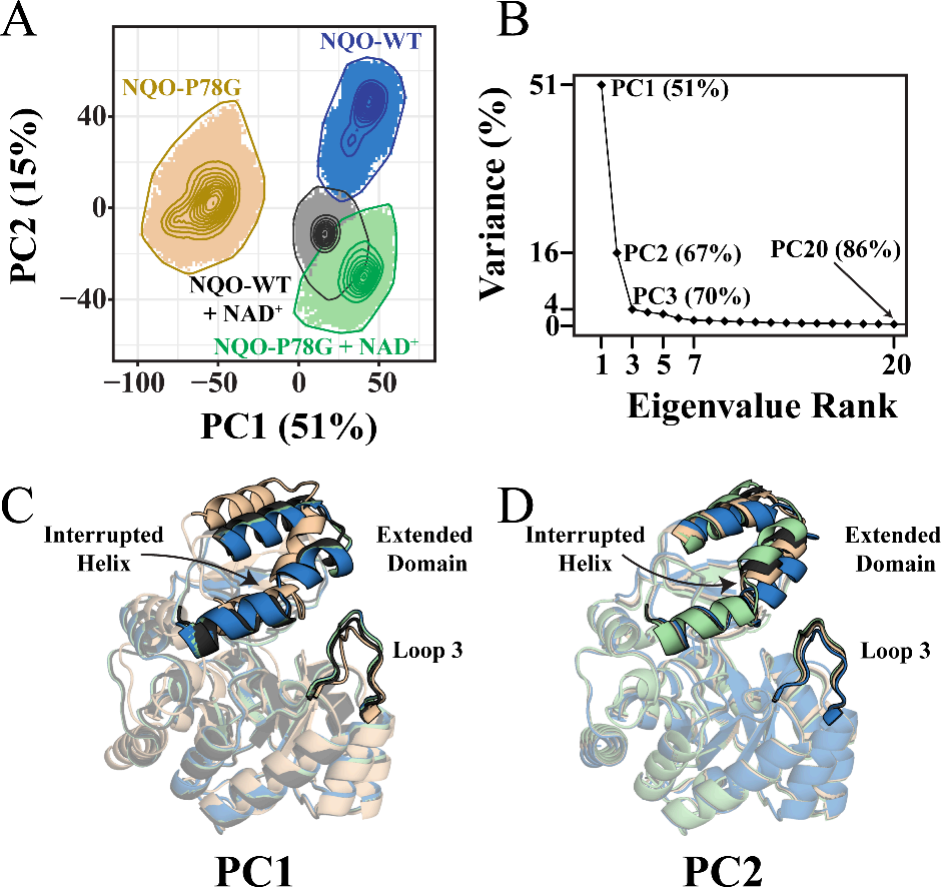
(A) A PCA contour plot depicts the conformations of NQO-P78G (tan), NQO-WT (blue), NQO-P78G with NAD^+^ (green), and NQO-WT with NAD^+^ (gray) sampled along the first two principal components (PC1 and PC2). The number in the axis label indicates the percent conformational variance captured by the corresponding principal component. The shaded regions depict potential conformations a system can adopt, while contour lines represent the probability density distributions of conformational states for each simulation. (B) A scree plot describing the compounded percent total variance captured as a function of eigenvalue ranks or principal components for NQO-P78G is shown. For example, the PC1 captures 51% of the total variance, while the first 20 principal components capture 86% of the total variance. The conformational state with the highest probability to be sampled for all four systems is shown at (C) PC1 or (D) PC2 where colors represent the same systems as described in panel (A).

The most probable conformation sampled from each simulation at PC1 or PC2 was projected onto the backbone of NQO to visualize how the P78G mutation altered the predominant configurations of the gate region and adjacent domains. The motions captured by PC1 describe conformational variation throughout the entire backbone of NQO, where the most significant deviations are observed in loop 3 and the extended domain (Figure 3C). In contrast, the motions captured by PC2 describe the structural variation surrounding the interrupted helix (Y^261^–Y^277^), which is a slightly unwound α-helix within the extended domain (Figure 3D). The PCA projections show that loop 3 and the extended domain are further displaced from one another in the ligand-free NQO-P78G simulation compared to the other simulations.

Variation in the NQO gate conformations was further investigated by determining the distance between the α-carbon atoms of Q80 on loop 3 and Y261 on the extended domain, as the two residues are thought to form a hydrogen bond that stabilizes the closed gate conformation.^29^ From the MD simulations, average separations of 21.0 ± 2.9 Å for NQO-P78G and 11.6 ± 1.4 Å for NQO-WT were measured between Q80 and Y261 (Figure 4).

**Figure 4:**
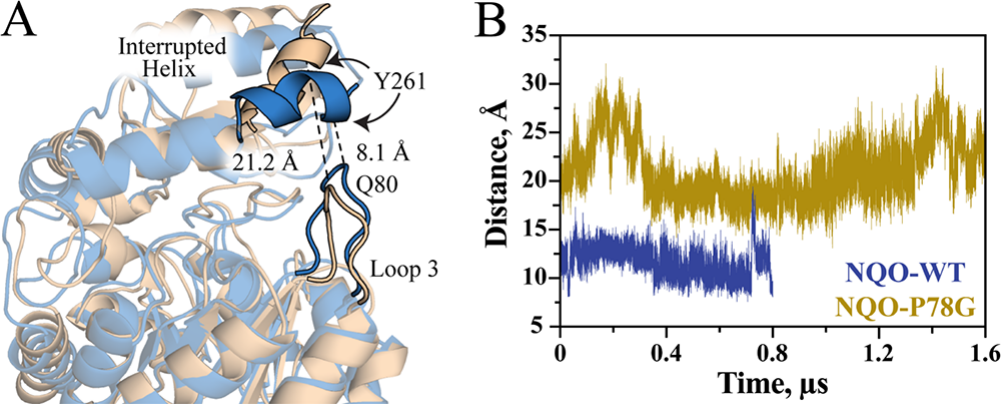
(A) The separation between the α-carbon atoms of Q80 and Y261 is visualized for ligand-free NQO-P78G (tan) and NQO-WT (blue). (B) The distance between the α-carbon atoms of Q80 and Y261 is shown for the last 0.8 and 1.6 μs for the ligand-free wild-type and mutant simulations, respectively, as these trajectories represent equilibrated backbone dynamics.

### Residue Contact Network Analysis

Differences in the residue–residue network communication pathways between ligand-free NQO-P78G and NQO-WT were determined via a dCNA to investigate how the P78G mutation altered interactions involving gate residues in NQO. Meaningful long-range residue contacts and the probabilities that they vary from wild-type to mutant in NQO were calculated, suggesting that the P78G mutation both broke and formed contacts throughout the structure. A dCNA community analysis was performed to investigate these potentially altered contacts further. This process subdivided the structure of NQO into 8 consensus communities, with each community representing a distinct structural portion of NQO characterized by more densely connected residues through stable contacts compared to contacts between other communities (Figure 5A). The region surrounding the entrance to the active site of NQO was subdivided into multiple communities, where the gray community contains the gate, the green community contains a portion of the interrupted helix, and the gold community contains the extended domain (Figure 5A). The total contact changes between each community were calculated and presented in a two-dimensional diagram, which revealed that the most significant contact change was a contact breakage with a difference of −7.8 between the gray and green communities (Figure 5B). The most significant contact changes between the gray, green, and gold communities were mapped onto the residues that underwent contact changes to better visualize the broken contacts found at this interface (Figure 5C).

**Figure 5:**
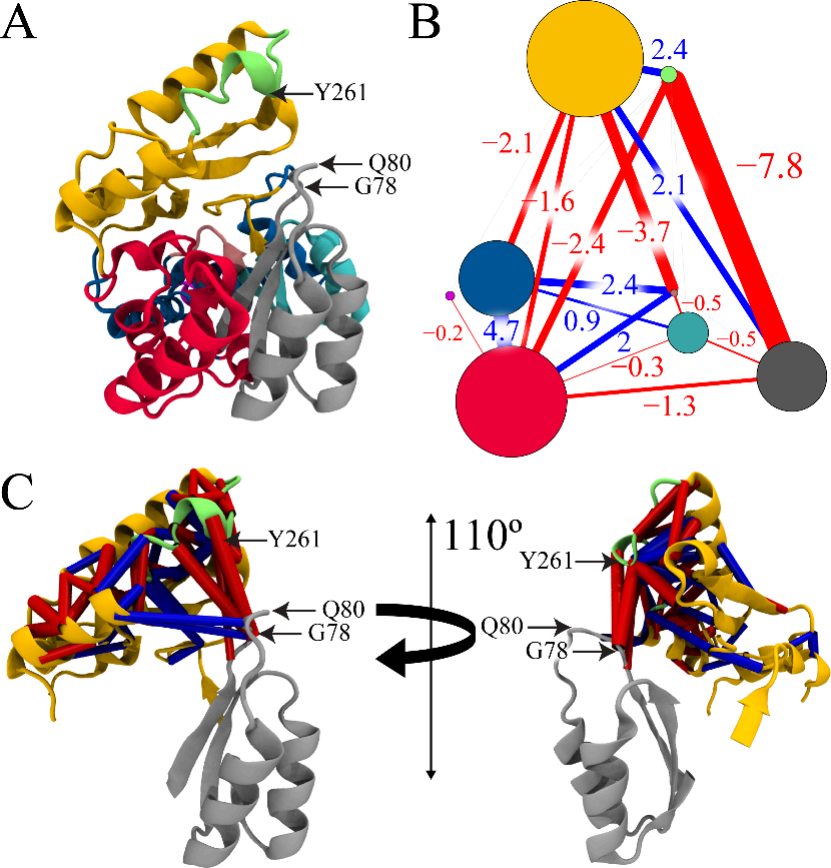
(A) The 8 consensus communities identified by the dCNA are color-coded onto the backbone of NQO-P78G, where the α-carbon atoms of G78, Q80, and Y261 are indicated via arrows. (B) Residue contact changes obtained from dCNA were mapped onto the community partition. Communities are represented by vertices that have the same color coding as in panel (A), whereas the radius of the vertices is proportional to the number of residues in that community. Contacts that form are depicted as blue lines, while contacts that break are depicted as red lines. The net contact probability changes (denoted by df) between two communities are labeled. (C) Residue–residue contacts with a |df| ≥ 0.5 were mapped onto the backbone of NQO-P78G containing only the gray, green, and gold communities to illustrate the interactions between loop 3 and the extended domain. Contacts that form are depicted as blue cylinders, and contacts that break are shown as red cylinders, where the cylindrical radius is proportional to |df|.

### Steady-State Kinetics of NQO-P78G

The steady-state kinetic parameters of NQO-P78G were determined and compared with NQO-WT to investigate how the altered gate dynamics affect the rate of substrate binding and turnover in NQO (Figure S1). CoQ_0_ was utilized as the oxidizing substrate at pH 6.0 to determine the kinetic parameters because substrate inhibition was observed with the previously employed oxidizing substrate benzoquinone.^25^ Structurally, CoQ_0_ consists of a benzoquinone core appended with two methoxy groups and a methyl group (Scheme 1). The estimated *K*_NADH_ and *k*_cat_/*K*_NADH_ values for NQO-WT are shown in Table 1; however, they are highly inaccurate due to substrate inhibition by NADH. Thus, the kinetic comparison between NQO-P78G and NQO-WT focuses on changes in the *k*_cat_, *K*_CoQ0_, and *k*_cat_/*K*_CoQ0_ values. The kinetic data of NQO-P78G and NQO-WT best fit to a ping-pong bi-bi steady-state kinetic mechanism, suggesting that the P78G mutation did not alter the overall steady-state mechanism of NQO. As shown in Table 1, the *K*_CoQ0_ and *k*_cat_ values decreased by 3.5-fold and 2-fold, respectively, for NQO-P78G compared to NQO-WT. In contrast, the *k*_cat_/*K*_CoQ0_ value increased by 1.8-fold for NQO-P78G with respect to NQO-WT.

**Table 1:** Steady-State Kinetic Parameters of NQO-WT and NQO-P78G^c^

kinetic parameter	NQO-WT	NQO-P78G
*k*_cat_, s^–1^	11 ± 1	5.4 ± 0.2
*K*_NADH_, μM	0.4 ± 0.3^a^	130 ± 10
*K*_CoQ0_, μM	35 ± 6	10 ± 1
*K*_is_, μM	1.1 ± 0.1	null^b^
*k*_cat_/*K*_NADH_, M^–1^ s^–1^	2.8 × 10^7^ ± 2.1 × 10^7^ ^a^	4.3 × 10^4^ ± 3.0 × 10^3^
*k*_cat_/*K*_CoQ0_, M^–1^ s^–1^	3.2 × 10^5^ ± 5.7 × 10^4^	5.6 × 10^5^ ± 4.6 × 10^4^
*R* ^2^	0.990	0.988

a Values are estimated and highly incorrect due to substrate inhibition by NADH.

b Value was not determined as the kinetic parameter was not applicable to the mechanism studied.

c Kinetic parameters were measured in 20 mM potassium phosphate, pH 6.0, and 100 mM sodium chloride at 25 °C by following the oxidation of NADH using the extinction coefficient for NADH at ε_340_ = 6220 M^–1^ cm^–1^.

### Reductive Half-Reaction with NADH

The rapid reaction kinetic parameters of NQO-P78G were investigated with NADH in a stopped-flow spectrophotometer at pH 6.0 and 25 °C and compared with NQO-WT to determine if the replacement of P78 with glycine altered the rate of flavin reduction and NADH binding affinity of NQO. The reductive half-reaction was monitored anaerobically as a previous oxidase activity assay revealed that NQO-P78G turns over with molecular oxygen at a reaction rate of 0.5 s^–1^ (data not shown), similar to NQO-WT.^25^ The stopped-flow traces for NQO-P78G were fit with a biphasic exponential process, where the first phase accounted for more than 90% of the total amplitude change and was assigned to flavin reduction (Figure 6A). In contrast, the slow phase accounted for less than 10% of the total absorbance change at 461 nm. The slow phase showed a *k*_obs_ value of 0.1 s^–1^ and was independent of the substrate concentration. A *k*_red_ value of 4.8 ± 0.3 s^–1^ and a *K*_d_ value of 450 ± 40 μM were measured for NQO-P78G upon reduction with NADH (Figure 6B). Qualitatively similar trends were observed in the stopped-flow traces with NQO-WT at pH 6.0, which yielded a *k*_red_ value of 12.9 ± 0.3 s^–1^ (Figure 6B). The 3-fold decrease in the *k*_red_ value from NQO-WT to NQO-P78G indicates that the rate of flavin reduction was affected by the P78G mutation. An accurate *K*_d_ value could not be determined for NQO-WT as it was not feasible to lower NADH concentrations below 90 μM while maintaining pseudo-first-order conditions.^24,25^ Nevertheless, NQO-WT could be saturated with 90 μM NADH, which suggests a *K*_d_ value of ≤5 μM. Thus, the replacement of P78 with glycine yielded at least an apparent 80-fold increase in the *K*_d_ value.

**Figure 6:**
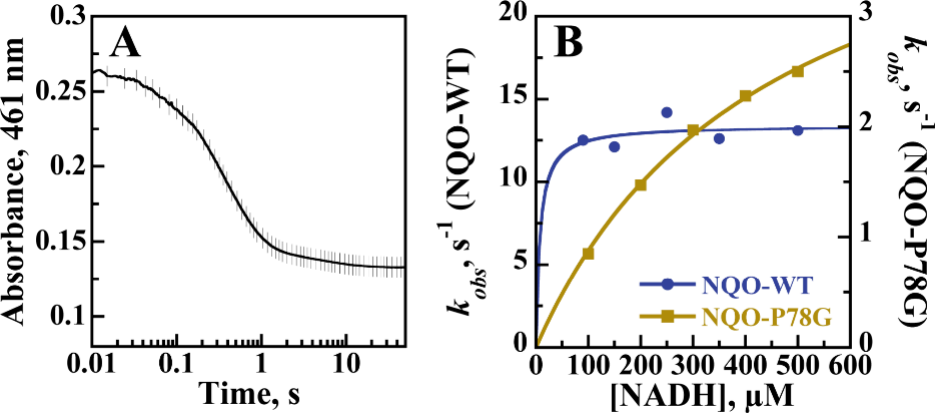
(A) Stopped-flow trace of NQO-P78G obtained upon anaerobically mixing with 100 μM NADH in 20 mM sodium pyrophosphate, pH 6.0, and 200 mM sodium chloride at 25 °C. Data were fit to eq 3. For clarity, every 10th experimental point is shown (vertical lines). (B) Observed rates of anaerobic flavin reduction (*k*_obs_) as a function of the NADH concentration for NQO-P78G (tan) and NQO-WT (blue). Data were fit to eq 4.

## Discussion

Recently, the catalytic role of Q80 in NQO was investigated by generating the mutants Q80G, Q80L, and Q80E.^24^ The reductive half-reactions with NQO-Q80 mutant enzymes and NADH revealed that disrupting the Q80-Y261 interaction increased the *K*_d_ value by ≥60-fold for Q80G and ≥25-fold for Q80L and Q80E. Interestingly, only NADH binding was significantly perturbed by the mutations, whereas the rate of flavin reduction by NADH was largely unaffected. The study proposed that the mutations destabilized NQO gate dynamics, which had a significant impact on the binding step with a minimal effect on other chemical steps in the catalytic cycle. Here, we build upon this previous NQO study by replacing the P78 residue in loop 3 with glycine to perturb gate dynamics and establish a link between conformational fluctuations and enzyme turnover in NQO from *P. aeruginosa* PAO1. As an initial assessment, the active site of NQO-P78G was probed using UV–visible absorption spectroscopy at pH values ranging from 8.0 to 11.5, which revealed no significant differences in oxidized FMN peak intensities and wavelengths at 370 and 461 nm between the mutant and wild-type NQO. These results demonstrate that the active site environment surrounding the flavin cofactor in NQO was minimally affected by pH and the P78G mutation.

Ligand-free MD simulations suggest that NQO-P78G samples more open gate conformations than NQO-WT, which would further expose the active site to the surrounding bulk solvent. The information used to support this hypothesis comes from PCA, distance calculations, and dCNA with NQO-P78G and NQO-WT in the ligand-free form. The PCA suggests that the changes in NQO dynamics induced by the P78G mutation primarily stem from loop 3 and the extended domain moving away from one another, which would allow the mutant enzyme to fluctuate between more open gate conformations compared to NQO-WT. This observation is supported by the distance calculations from MD simulations that suggest that the separation between gating residues Q80 and Y261 is, on average, 9.4 Å larger in NQO-P78G relative to NQO-WT (Figure 4). Furthermore, the dCNA indicates that the greatest contact breakages stemming from the P78G mutation are between the backbone surrounding P78 and residues surrounding Y261 within the interrupted helix. This observation suggests that substituting P78 with glycine in NQO produces more open gate conformations by disrupting residue–residue contacts between loop 3 and the extended domain. Previously, a structural study on NQO proposed that loop 3 dynamics alone act as the gate to seal off the active site from the bulk solvent by facilitating an interaction between Q80 and a structurally static Y261.^29^ However, the computational analyses discussed here suggest that the gate region in NQO comprises an extended domain that fluctuates in tandem with loop 3.

To form the NQO-NAD^+^ complex, NQO-P78G may undergo more extensive conformational changes throughout the entire protein scaffold compared to NQO-WT. Evidence to support this conclusion comes from the PCA data for NQO-P78G and NQO-WT in both the ligand-free and NAD^+^-bound forms. Backbone conformations for both the mutant and wild-type NAD^+^-bound simulations are similar at PC1 and PC2, suggesting that the dynamics of the enzyme–product complex were largely unaffected by the P78G mutation. Comparing the conformational states of the NAD^+^-bound complexes to those sampled by ligand-free NQO-WT reveals a significant structural overlap along PC1 and only slight variations along PC2, primarily due to fluctuations at the interrupted helix. In contrast, the preferred conformational states of ligand-free NQO-P78G do not overlap with the NAD^+^-bound simulations along either PC1 or PC2. This observation suggests that NQO-P78G must undergo significant conformational changes across the TIM-barrel domain, extended domain, and interrupted helix to form the ligand-bound complex during the substrate-binding step. In contrast, NQO-WT only requires slight conformational changes at the interrupted helix to sample the ligand-bound conformations, thus demonstrating that the NQO-NAD^+^ complex may be more accessible compared to NQO-P78G.

The proposed more open gate conformations sampled by NQO-P78G do not alter the rate-limiting step in NQO, which is the reduction of enzyme-bound flavin through a hydride transfer from NADH. Evidence to support this conclusion comes from the anaerobic reductive half-reactions with NADH and steady-state kinetics determined by varying the concentrations of NADH and CoQ_0_ for NQO-P78G and NQO-WT at pH 6.0 and 25 °C. For NQO-P78G, a *k*_cat_ value of 5.4 ± 0.2 s^–1^ and a *k*_red_ value of 4.8 ± 0.3 s^–1^ were measured. In comparison with NQO-WT, a *k*_cat_ value of 11.0 ± 1.0 s^–1^ and a *k*_red_ value of 12.9 ± 0.3 s^–1^ were measured. The observed differences between *k*_cat_ and *k*_red_ values are nominally 12 and 16% for NQO-WT and NQO-P78G, respectively, which is consistent with the rate of flavin reduction being almost entirely rate-limiting in both enzymes. Directly comparing the *k*_cat_ and *k*_red_ values between NQO-P78G and NQO-WT yielded 2-fold and 3-fold differences, respectively, suggesting that the hydride transfer step in NQO was minimally affected by the P78G mutation. The observations reported here are consistent with mechanistic investigations on NQO-Q80 mutants, which observed differences of 4–15% between *k*_cat_ and *k*_red_ values.^24^ Thus, the mechanistic investigations demonstrate that altering the gate fluctuations does not change the ping-pong mechanism and indicate that once the enzyme–NADH complex forms and the FMN cofactor is reduced, NQO-P78G functions as well as NQO-WT.

The replacement of P78 with glycine minimally increased the rate of association of the oxidized quinone with the reduced enzyme to form enzyme–quinone complexes that partition forward to catalysis. Evidence to support this conclusion comes from the steady-state kinetics with NADH and CoQ_0_ for NQO-P78G and NQO-WT at pH 6.0 and 25 °C. A 2-fold increase in the *k*_cat_/*K*_CoQ0_ value for NQO-P78G was observed compared to NQO-WT. Typically, mutations that replace conserved residues in enzymes yield lower *k*_cat_/*K*_m_ and *k*_cat_ values.^57,58^ Therefore, the unexpected increase in the *k*_cat_/*K*_CoQ0_ value implies that the P78G mutation minimally increased the rate of quinone capture, which is defined as all the kinetic steps starting from free enzyme and substrate up to and including the first irreversible step. Similarly, the NQO-Q80E mutation minimally increased the *k*_cat_/*K*_m_ values by 1.1- to 1.5-fold with the quinones CoQ_0_, 1,4-benzoquinone, toluquinone, and juglone.^24^ A rational explanation for these observations is that the P78G and Q80E gate mutations destabilized interactions between loop 3 and the extended domain, thus increasing the exposure of the active site to the bulk solvent in NQO. This increased solvent exposure allows CoQ_0_ to access the active site pocket more easily and subsequently form the interactions necessary for a stable enzyme–quinone complex.

The binding affinity of NADH for NQO was significantly decreased by the replacement of P78 with glycine. Evidence to support this conclusion comes from the anaerobic reductive half-reactions of NQO-P78G and NQO-WT with NADH. An 80-fold increase in the *K*_d_ value was estimated from NQO-WT to NQO-P78G, suggesting that the P78G mutant has a decreased affinity for NADH. This reduced affinity can be explained by the NAD^+^-bound structure of NQO (PDB: 6E2A), which reveals that loop 3 and the extended domain form multiple interactions to stabilize the ribose, pyrophosphate, and adenine moieties of NAD^+^ near the entrance of the active site.^29^ Specifically, the backbone carbonyl of P78 from loop 3 forms a hydrogen bond with the 2′-hydroxyl of the adenine ribose, while the backbone amide of Q80 from loop 3 and G270 from the extended domain hydrogen bond to the O1A and O2A atoms of the adenine phosphate, respectively. The extended domain residue Y261 also plays an important role in binding the pyrimidine nucleotide, as it forms a π-stacking interaction with the adenine rings. Thus, the estimated increase in the *K*_d_ value for NQO-P78G with NADH likely results from the more open gate conformations sampled by loop 3 and the extended domain, which prevents key interactions involved in NADH binding from forming. This conclusion is corroborated by the PCA and distance calculations that demonstrate that NQO-P78G would have to undergo extensive conformational changes to sample NAD^+^-bound conformations, especially at the gate region.

Decreasing gate rigidity through a P78G mutation allows NQO to accommodate the smaller substrate, CoQ_0_, slightly better while simultaneously inhibiting the bulkier substrate, NADH, from properly forming the hydrogen bonds necessary to achieve a stable ligand-bound complex. Evidence to support this conclusion comes from PCA, dCNA, steady-state kinetics determined with NADH and CoQ_0_, and anaerobic reductive half-reactions with NADH. The primary impact of the P78G mutation was a decrease in gate rigidity, which broke interdomain interactions and exposed the active site of NQO to the bulk solvent, as proposed by PCA and dCNA. Steady-state kinetics indicated that this increased active site exposure improved the access of CoQ_0_ to the active site of NQO, as NQO-P78G minimally enhanced the rate of enzyme–quinone complex formation. In contrast, the anaerobic reductive half-reactions with NQO-P78G and NADH revealed that the increased active site exposure reduced the affinity of the enzyme for NADH. A major difference between these two substrates is their size, where NADH is much larger than CoQ_0_ and requires binding interactions from both active site and gate residues to form a stable ligand-bound complex. These observations suggest that the reduced gate rigidity in NQO-P78G ultimately altered substrate binding by enhancing the accessibility of active site residue interactions while perturbing the gate residue interactions. As a result, the binding affinity increased for substrates that mainly form active site residue interactions and decreased for those that require gate residue interactions.

## Conclusions

In summary, this investigation directly builds upon prior NQO studies by demonstrating that decreasing gate rigidity alters substrate binding in a similar, if not equivalent, capacity to disrupting the Q80-Y261 hydrogen bond that stabilizes the closed gate conformation. This conclusion is evidenced by the comparable changes in *k*_cat_/*K*_M_ values with quinone substrates and apparent *K*_d_ values with NADH between the P78G and Q80 mutants. Moreover, this study provides an in-depth example detailing how enzyme dynamics can be just as crucial as key side chain interactions when forming enzyme–substrate complexes. We hope that by highlighting the potential benefits of altering gate physicochemical properties, this study will encourage future enzymatic studies aimed at modulating kinetic parameters and protein function to account for gate dynamics.

## Supplementary Material



## Notes

Steady-state kinetic data of NQO with CoQ_0_ as a substrate (Figure S1) (PDF)

## Notes

NQO: UniProt Accession ID Q9I4V0; Enzyme Commission Accession ID 1.6.5.9.

## Abbreviations

NQONADH: quinone oxidoreductase
MD: molecular dynamics
PCA: principal component analysis
PC: principal component
RMSD: root-mean-square deviation
dCNA: difference contact network analysis
CoQ_0_: coenzyme Q0
