# Supplementary material for: Mechanistic and Molecular Dynamics Studies Reveal that Increased Loop 3 Mobility Alters Substrate Capture in an NADH:Quinone Oxidoreductase
Source: Biochemistry. 2025 Dec 13;65(1):104–13. doi: 10.1021/acs.biochem.5c00559 (PMC12781114; doi:10.1021/acs.biochem.5c00559)
Supplement: Supplementary file 1 [file bi5c00559_si_001.pdf]

Supporting Information

Mechanistic and molecular dynamics studies reveal that increased loop 3 mobility alters substrate capture in an NADH:quinone oxidoreductase

Benjamin D. Dratch[α], *Daniel Ouedraogo*[α], *Jacob Ball*[α], *Donald Hamelberg*[α,γ*], *and Giovanni Gadda*[α β, γ*]

[α]Departments of Chemistry, [β]Biology, and [γ]The Center for Diagnostics and Therapeutics, Georgia State University, Atlanta, GA 30302-3965

*Correspondence: ggadda@gsu.edu

Table of Contents

Supplementary Figures 2

Supplementary Figures

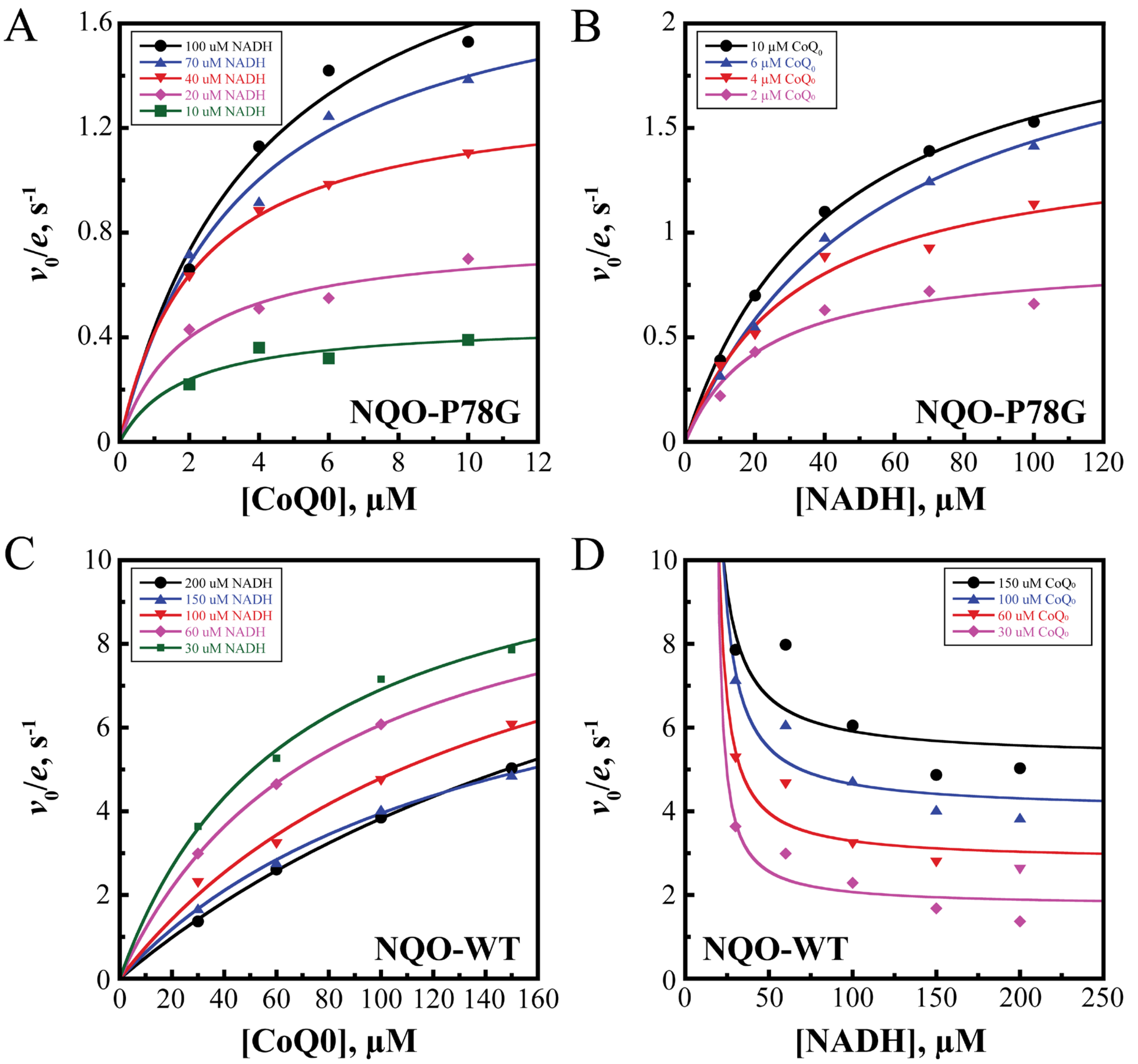

Figure S1. Steady-state kinetics of NQO with CoQ_0 as a substrate. Michaelis-Menten plots for (**A, B**) NQO-P78G and (**C, D**) NQO-WT. The initial rate of reaction was measured in 20 mM potassium phosphate, pH 6.0, and 100 mM sodium chloride at 25 °C. Data for NQO-P78G and NQO-WT were fit to Equation 1 and 2, respectively.
